# Structure and ligand binding of the glutamine-II riboswitch

**DOI:** 10.1093/nar/gkz539

**Published:** 2019-06-19

**Authors:** Lin Huang, Jia Wang, Andrew M Watkins, Rhiju Das, David M J Lilley

**Affiliations:** 1 Cancer Research UK Nucleic Acid Structure Research Group, MSI/WTB Complex, The University of Dundee, Dow Street, Dundee DD1 5EH, UK; 2 Department of Biochemistry, Stanford University School of Medicine, Stanford, CA 94305, USA

## Abstract

We have determined the structure of the glutamine-II riboswitch ligand binding domain using X-ray crystallography. The structure was solved using a novel combination of homology modeling and molecular replacement. The structure comprises three coaxial helical domains, the central one of which is a pseudoknot with partial triplex character. The major groove of this helix provides the binding site for L-glutamine, which is extensively hydrogen bonded to the RNA. Atomic mutation of the RNA at the ligand binding site leads to loss of binding shown by isothermal titration calorimetry, explaining the specificity of the riboswitch. A metal ion also plays an important role in ligand binding. This is directly bonded to a glutamine carboxylate oxygen atom, and its remaining inner-sphere water molecules make hydrogen bonding interactions with the RNA.

## INTRODUCTION

Riboswitches are *cis*-acting regulatory elements often found in the 5′-untranslated regions (UTR) of bacterial mRNA (1–3). They usually bind cellular metabolites resulting in up- or down-regulation of the downstream gene at the transcriptional or translational level. More than 40 classes of riboswitch are known, which respond to a variety of metabolites including co-enzymes, nucleobases, amino acids and even single ions, and in some cases different classes of riboswitch exist that respond to the same metabolite, with different structures and sometimes different regulatory mechanisms.

Regulation of nitrogen assimilation (4,5) is particularly important in marine bacteria such as the photosynthetic cyanobacteria, where the key enzyme is glutamine synthetase (GS) that condenses glutamate with ammonia to form glutamine (6). This is subject to several forms of regulation, including the transcriptional regulator NtcA and the GS-inactivating protein factors IF7 and IF17, encoded by *gif*A and *gif*B, respectively (7,8). Ames and Breaker (9) identified two ∼60 nt elements in the 5′-UTRs of genes involved in nitrogen metabolism in cyanobacterial and metagenomic sequences. One is based on a three-way helical junction with a loop E motif, termed *gln*A, and the other with a pseudoknot termed the downstream peptide (DP) motif. Using in-line probing they demonstrated that the RNA binds L-glutamine with mM affinity, but discriminated against a series of similar compounds. Although the two elements have distinct secondary structures, Ames and Breaker (9) showed the sequences could be arranged to reveal a common arrangement to some degree. Klähn *et al.* (10) demonstrated that the elements acted as riboswitches that control the expression of IF17 synthesis. They suggested that the *gln*A and DP riboswitches be renamed glutamine-I and -II, respectively. Using a GFP fusion, they showed that the glutamine-II riboswitch acts as an ON switch to control the synthesis of the GS regulatory protein. Given the proximity to the initiator codon (Supplementary Figure S1), it is likely to act as a translational riboswitch.

The structure of the glutamine-I riboswitch ligand binding domain was solved using X-ray crystallography by Ren *et al.* (11). This showed the fold of the helical junction and the manner of binding of the L-glutamine ligand. In this work we have solved the crystal structure of the glutamine-II riboswitch, revealing how ligand specificity is achieved. The structure suggests how the riboswitch could be divided into two separate RNA molecules, and we show that addition of L-glutamine induces the formation of a functional ligand binding domain.

## MATERIALS AND METHODS

### RNA synthesis

The glutamine-II riboswitch wild-type has the sequence from *Prochlorococcus sp*. RS01 genome (all sequences written 5′ to 3′):

CGUUCAUCCAAGUUUAUAUCUCUGGACGCAUGAAAUGGGAGUAGGGAACGGGAUUCUCAU

The RNA used for crystallization was (^Br^C = 5-bromocytidine):

CGUUCAC(^Br^C)(^B^rC)UUCGGGGCGCAUGAAAUGGGAGUAGGGAACGGGAUUCUCAU

The RNA used for calorimetry was:

CGUUCACCCUUCGGGGCGCAUGAAAUGGGAGUAGGGAACGGGAUUCUCAU

RNA oligonucleotides were synthesized using ***t***-BDMS phosphoramidite chemistry (12) as described in Wilson *et al.* (13), implemented on an Applied Biosystems 394DNA/RNA synthesizer. RNA was synthesized using ribonucleotide phosphoramidites with 2′O-*tert*-butyldimethyl-silyl (*t*-BDMS) protection (14,15) (Link Technologies). All oligoribonucleotides were redissolved in 100 μl of anhydrous DMSO and 125 μl of triethylamine trihydrofluoride (Sigma-Aldrich) to remove *t*-BDMS groups, and agitated at 65°C in the dark for 2.5 h. After cooling on ice for 10 min, the RNA was precipitated with 1 ml of butanol, washed twice with 70% ethanol and suspended in double-distilled water.

RNA was further purified by gel electrophoresis in polyacrylamide under denaturing conditions in the presence of 7 M urea. The full-length RNA product was visualized by UV shadowing. The band was excised and electroeluted using an Elutrap Electroelution System (GE Healthcare) into 45 mM Tris-borate (pH 8.5), 5 mM EDTA buffer for 8 h at 200 V at 4°C. The RNA was precipitated with ethanol, washed once with 70% ethanol and suspended in water or ITC buffer (40 mM HEPES-K (pH 7.0), 100 mM KCl, 10 mM MgCl_2_).

### Crystallization, structure determination and refinement

A solution of 0.8 mM glutamine-II riboswitch RNA (50 nt) in 5 mM HEPES (pH 7.6), 100 mM KCl was heated to 95°C for 1 min. The solution was slowly cooled to 20°C and MgCl_2_ added to a final concentration of 5 mM. L-glutamine (Sigma-Aldrich) was added to a final concentration of 5 mM. Crystals were grown by sitting drop vapor diffusion at 7°C using drops prepared by mixing 1 μl of the RNA–ligand complex with 1 μl of a reservoir solution comprising 100 mM sodium cacodylate (pH 6.0), 200 mM Mg acetate and 19% (v/v) PEG 8000. Crystals appeared after 7 days. The crystals were transferred into 50 mM sodium cacodylate (pH 6.0), 100 mM Mg acetate, 3.5 M Na formate and 10% v/v PEG 8000 for 1 h, then flash frozen by mounting in nylon loops and plunging into liquid nitrogen.

Diffraction data were collected on beamline I03 of Diamond Light Source (Harwell, UK). Data were processed by XIA2 (16). The resolution cutoff for the data was determined by examining by CC1/2 and the density map (17). The structure was determined by molecular replacement using PHASER (18) and the search model that was derived by homology modeling, as described below.

Crystals grew in space group C222_1_ with unit cell dimensions *a* = 76.5 Å, *b* = 115.7 Å and *c* = 80.6 Å. From crystal density considerations (19,20), two RNA molecules were expected to be present in the asymmetric unit. The search model contained the P2 and the PK pseudoknot helix of the structure derived by homology modeling. The best solution gave translation function *Z*-scores of 7.6 and 14.9 for one and two copies, respectively, and an overall log likelihood gain (LLG) of 345. Another structure model of the P1 (3 base pairs with a UNCG loop was derived from PDB: 1F7Y) was used for the second-round search. The top solution gave translation function *Z*-scores of 11.3, and an overall LLG of 464. Inspection of the electron density and crystal packing revealed that only one P1 was placed correctly. After correction *R*_free_ reduced smoothly to 35% during refinement. The resulting electron-density maps revealed the remaining RNA density, and were built *de novo* on the basis of the difference map. Models were adjusted manually using Coot (21) and subjected to several rounds of adjustment and optimization using Coot, phenix.refine and PDB_REDO (22). Composite omit maps were calculated using PHENIX. Model geometry and the fit to electron-density maps were monitored with MOLPROBITY (23) and the validation tools in Coot. Atomic coordinates and structure factor amplitudes have been deposited with the PDB with accession code 6QN3. Crystallographic statistics are presented in Table 1.

**Table 1.** Details of data collection and refinement statistics for the crystallographic data as deposited with the PDB

PDB	6QN3
**Data collection**	
Space group	C222_1_
Cell dimensions	
*a, b, c* (Å)	76.5, 115.7, 80.6
*α, β, γ* (°)	90 90 90
Wavelength	0.9195
Resolution (Å)	80.58–2.20 (2.24–2.20)
*R*_merge_	0.101 (1.639)
*R*_pim_	0.044 (0.762)
*I* / σ*I*	8.7 (1.0)
CC (1/2)	1.00 (0.59)
Completeness (%)	100 (99.6)
Redundancy	6.4 (5.6)
**Refinement**	
Resolution (Å)	47.00–2.30
(2.37–2.30)	
No. of reflections	30523(2646)
*R*_work_ / *R*_free_	0.249/ 0.297 (0.406/0.422)
No. of atoms	
Macromolecules	2160
ligands	8
*B*-factors	
Macromolecules	79.44
ligands	76.22
R.m.s. deviations	
Bond lengths (Å)	0.008
Bond angles (°)	1.51
Coordinate error (Å) (Maximum-likelihood based)	0.44

Values in parentheses are for highest resolution shell.

### Homology modeling

Homology modeling was conducted with Rosetta 3.8 (24). First, nucleotides A:1–11, A:16–22, A:55–59 and E:101 were excised from the deposited PDB structure 5DDP using a text editor. These residues represent the portion of the glutamine-I riboswitch that is well conserved with respect to glutamine-II riboswitch. Those 5DDP nucleotides were renumbered to A:3–14, A:22–28, A:48–52 and B:101 to correspond to the glutamine-II riboswitch sequence:

renumber_pdb_in_place.py start.pdb A:3-14 A:22-28 A:48-52 B:101

A specially formatted FASTA file was constructed to specify the target full RNA sequence for modeling:

> glutamine_II A:1-62 B:101

ucacguucaccucguuuucagcgaggcgcaguucgacucaggccauggaacggggaccugagQ

Two ideal A-form helices serve as a starting model for the P2:

rna_helix.py -seq cucagg ccugag

mv helix.pdb helix_1.pdb

renumber_pdb_in_place.py helix_1.pdb A:37-42 A:57-62

rna_helix.py -seq cc gg

mv helix.pdb helix_2.pdb

renumber_pdb_in_place.py helix_2.pdb A:43-44 A:54-55

Finally, a stepwise Monte Carlo simulation was conducted with these inputs.

stepwise -s helix_1.pdb helix_2.pdb start.pdb -fasta target.fasta

-motif_mode -score:weights stepwise/rna/rna_res_level_energy4.wts

-restore_talaris_behavior -cycles 2000 -nstruct 500

The 10 best scoring models were inspected in order to yield five top candidates; the pairwise RMSD between all models was computed, and the five models with the highest average mutual RMSD were chosen (Supplementary Table S1).

### Isothermal titration calorimetry

Titrations were performed at 298 K using an ITC-200 microcalorimeter (GE). RNA solutions (100–300 μM) were prepared by diluting concentrated stocks into the binding buffer containing 40 mM HEPES-K (pH 7.0), 100 mM KCl, 10 mM MgCl_2_ (ITC buffer). L-glutamine was prepared in the same binding buffer with a concentration of 2–4 mM. Solutions were degassed for 2–5 min before loading. The sample cell was filled with 200 μl of RNA. L-glutamine was injected in a volume of 0.4 μl for the first injection and 2 μl for the next 19 injections using a computer-controlled 40 μl microsyringe with an injection interval of 120 s. Titration of ligands into the binding buffer or titration of the binding buffer into the RNA solution resulted in negligible evolution of heat. Integrated heat data were analyzed using a one-set-of-sites model in MicroCal Origin following the manufacturer’s instructions. The first data point was excluded in analysis. The binding parameters enthalpy Δ*H* (cal mol^−1^), association constant *K*_a_ (M^−1^) and *n* (bound ligands per RNA) were variables in the fit. The binding free energy Δ*G* and reaction entropy Δ*S* were calculated using the relationships Δ*G*  =  −*RT* ln *K*, where *R* = 1.987 cal mol^−1^ K^−1^, *T* = 298 K and Δ*G* = Δ*H* − TΔ*S*. The dissociation constant *K*_d_ was calculated as 1/*K*_a_.

### Sequence alignment and analysis

Glutamine-II riboswitch sequences were taken from Rfam under accession RF01704. The sequences were manually realigned against the crystal structure using Jalview (25). All sequence composition and covariation analysis were calculated by Jalview.

## RESULTS

### Construction and crystallization of the glutamine-II riboswitch

The glutamine-II riboswitch from the *Prochlorococcus sp*. RS01 genome was selected for crystallization after comparison of sequences. To facilitate crystallization and structure determination, the P1 region of the wild-type sequence AUCCAAGUUUAUAUCUCUGGAC was changed to AC(^Br^C)(^Br^C)UUCGGGGC (^Br^C = 5′bromocytosine). Crystals grew in space group C222_1_ with unit cell dimensions *a* = 76.5 Å, *b* = 115.7 Å and *c* = 80.6 Å, and diffracted to 2.2 Å resolution. Our intention was to solve the structure on the basis of the anomalous scatter of bromine by SAD. However, this proved not to be possible using Autosol in PHENIX, AUTOSHARP and CRANK2 as a result of the anisotropy and high R-merge of the data. The structure was therefore determined by molecular replacement using PHASER (18) and a search model derived by Rosetta homology modeling using the stepwise Monte-Carlo algorithm (24) (see ‘Materials and Methods section’). The data were refined at 2.3 Å resolution. A composite omit map of the riboswitch is shown in Supplementary Figure S2. The quality of the map is lowered by the anisotropy in the diffraction. The quality of the electron density is better in some regions than in others. One asymmetric unit contains two RNA molecules, and in general the quality of the density map is better for chain A than for chain B.

### The structure of the glutamine II-riboswitch

**Figure 1.**
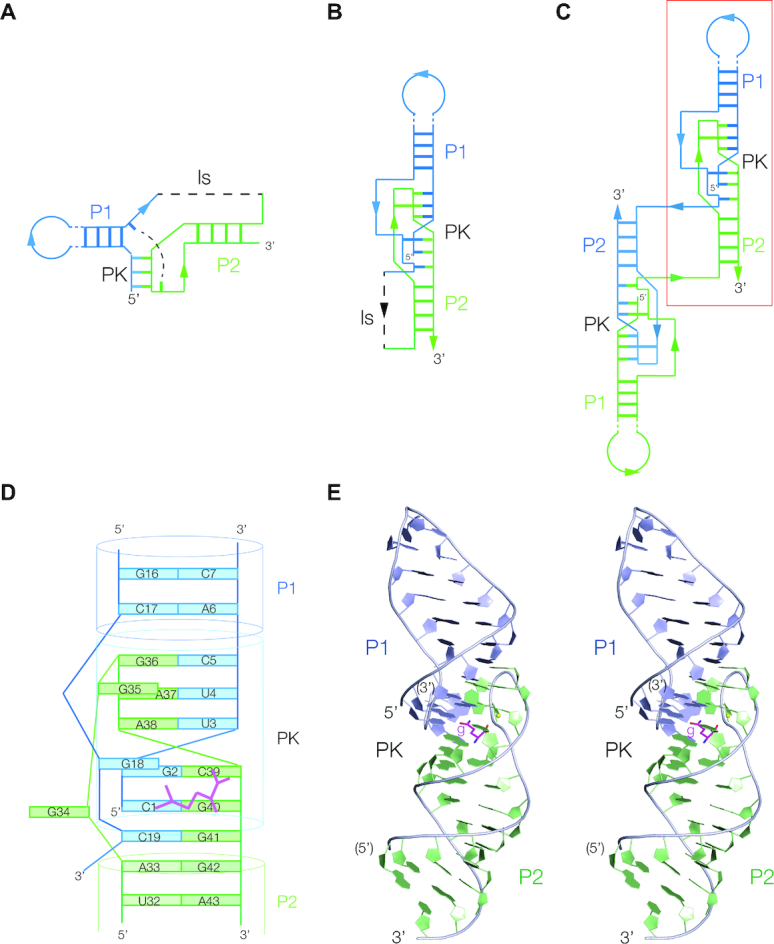
The overall structure and organization of the glutamine-II riboswitch. In the schematic diagrams, the individual strands are colored blue and green. The helices are designated P1, P2 and PK, and the linking strand ls. (**A**) The secondary structure depicted in the manner used by Ames and Breaker (9). The long-range C19:G41 base pair is indicated by the broken line. (**B**) The functional unit of the riboswitch found in the crystal. The broken line shows the expected connectivity when the RNA folds from a single polynucleotide chain. (**C**) The asymmetric unit found in the crystal, comprising two complete riboswitches, with an exchange of strands. The structure is shown in Supplementary Figure S3. An individual functional riboswitch is boxed in red. (**D**) A schematic of the structure of the functional core of the riboswitch. A glutamine molecule is indicated in magenta. (**E**) The structure of the complete riboswitch functional unit, shown in parallel-eye stereoscopic representation. Nucleotides are colored blue and green in the same manner as the schematics, and the glutamine (‘g’) is shown in stick form, colored magenta.

The structure of the functional unit of the riboswitch in the crystal comprises three coaxial helical segments: P1 – PK – P2 (Figure 1). This has been recolored in Supplementary Figure S3 to emphasize the three coaxial segments that comprise the structure. The 5′ end of the RNA lies within the PK helix, continues through the P1 stem–loop, then becoming the linking strand is that forms a triple interaction in PK (see below). The ls strand makes the long-range C19:G41 base pair at the lower end of PK before connecting at the 5′ end of P2. It then rises up P2 before forming the pseudoknot loop, making the G35>A37:U4 base triple at the top and then runs down as the second strand of PK. It finally forms the second strand of P2 before reaching its 3′ terminus.

P1 and P2 adopt regular A-form RNA duplexes, except for the nucleotides that adjoin the PK helix. The nucleobases in the A6:C17 pairing adjacent to the PK helix are coplanar but not hydrogen bonded, and a *cis*-Watson–Crick A33:G42 base pair forms at the end of P2 at the intersection with PK. P1 is a stem–loop capped by a UUCG tetraloop. This is an example of the common UNCG type of stable tetraloop (26). U10 and G13 are coplanar and connected by hydrogen bonds from G13 N1 to U10 O2, and U10 O2’ to G13 O6. There is a sharp turn at U11, with a hydrogen bond from U11 O2’ to G13 N7, and the ribose of C12 is stacked on the nucleobase of G13.

**Figure 2.**
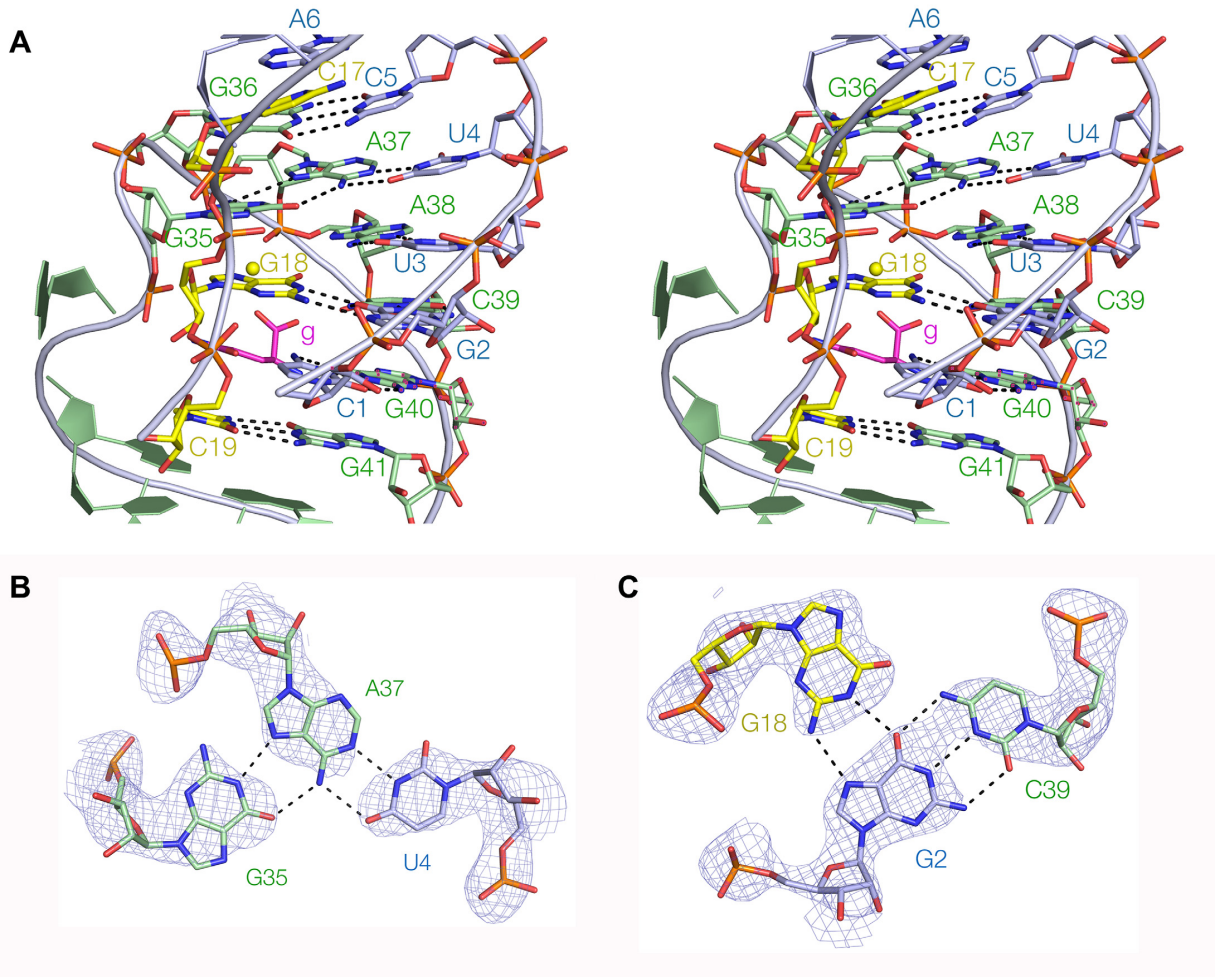
The structure of the PK pseudoknot helix. (**A**) A parallel-eye stereoscopic representation of the complete PK helix. C17, G18 and C19 of the ls strand (colored yellow here for clarity) are located in the major groove of the PK helix, and G18 contributes to the G18>G2:C39 triple interaction. (**B**) The structure of the G35>U4:A37 triple interaction. (**C**) The structure of the G18>G2:C39 triple interaction. In parts (**B**) and (**C**), the composite omit map of electron density is shown contoured at 2 σ.

The PK helix is located between P1 and P2, and has a more complex structure (Figure 2). It is the pseudoknot interaction in the structure, with partial triplex character. While the PK helix is fundamentally double-stranded, the ls strand (colored yellow in this figure for clarity) is located within its major groove, forming two important interactions. One is a G18 > G2:C39 base triple (Figure 2C) with two hydrogen bonds between G18 N1 and N2 with G2 O6 and N7, respectively, on its Hoogsteen edge. In addition, the ls strand makes a conserved long-range base pair (predicted by Ames and Breaker (9) on the basis of phylogenetic comparisons) between C19 and G41, forming the final base pair of PK before the start of P2. Thus the PK pseudoknot helix comprises base pair (G36:C5), triple (G35>A37:U4), base pair (A38:U3), triple (G18>C39:G2), base pair (G40:C1) and finally the long-range base pair (G41:C19). This region is the functional heart of the riboswitch, creating the glutamine binding site as we discuss below.

### The structure of the glutamine-II riboswitch in the crystal

Although what we describe above is clearly the functional unit of the riboswitch, in the crystal this is made up of two RNA molecules (colored here blue and green) (Figure 1C and Supplementary Figure S4). The RNA has dimerized during the crystallization process, and each unit comprises P1 from one molecule, P2 from the other and a hybrid PK helix. This involves the formation of intermolecular base pairs and triple interactions. The two units are connected through the exchange of strands at the 5′ end of P2 and the ls strand at the C19:G41 base pair. The exchanging strands comprise five nucleotides, with no hydrogen bonding but extensive stacking of the nucleobases. To form a monomeric species (the presumed active form of the riboswitch in the cell), the then-intramolecular linker will be required to connect two positions within a single RNA molecule that are separated by 24 Å, which is clearly possible. We have generated a model of the presumed monomer that shows that the linker readily connects the P1 and P2 segments (Supplementary Figure S5). Such domain exchange within a crystal lattice is not unusual in small RNA molecules, having been observed in the ZTP riboswitch (27). This was also found in the VS ribozyme (28) where the formation of a functional hybrid active site had been previously shown to occur in solution (29).

### Ligand binding in the glutamine-II riboswitch

**Figure 3.**
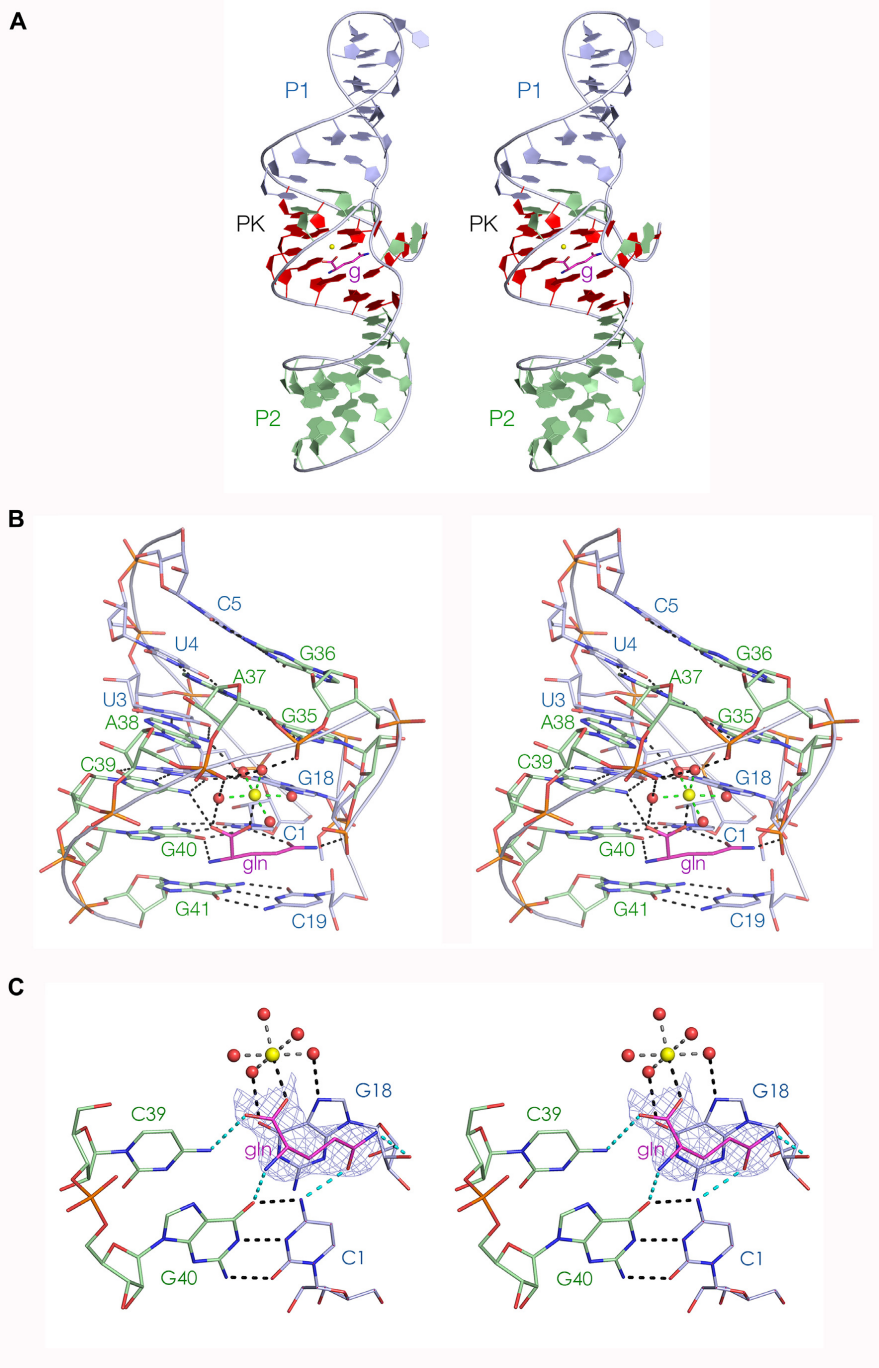
The ligand binding site in the glutamine riboswitch. Parallel-eye stereoscopic representations are shown for each view. (**A**) The location of highly conserved nucleotides. Nucleotides that are more than 97% conserved are colored red. Note that these are clustered in the PK helix, and surround the glutamine ligand (‘g’, shown in stick form colored magenta). (**B**) The glutamine binding site in the PK helix. This view is from the opposite side compared to that in Figure 2A. (**C**) Close-up view of the binding site of the glutamine-II riboswitch, with the composite omit map for the bound glutamine contoured at 1.5 σ. Diagram of the ligand binding site showing an omit map for the hydrated metal ion is shown in Supplementary Figure S7. Hydrogen bonds between the glutamine ligand and the RNA are highlighted cyan. The metal ion is shown as the yellow sphere, and the inner sphere of hydration water molecules as red spheres. Note that G2 of the G2>G18:C39 triple interaction has been undisplayed for clarity in this view.

Ames and Breaker (9) identified 11 nucleotides that were > 97% conserved. Guided by the crystal structure we realigned the section linking P2 and PK, leading to the addition of one more highly conserved nucleotide (Supplementary Figure S6). The realignment then leads to the important *cis*-Watson–Crick base pair A33:G42 having a conservation of 99.5%. This base pair is important in making the transition from the PK to the P2. When we color our crystal structure so that all the conserved nucleotides are highlighted red (Figure 3A), we find that these to be clustered, exclusively contained within the PK pseudoknot region. In fact the only nucleotides within the PK region that are not highlighted are the four that comprise the two base pairs adjacent to P2, i.e. U4:A37 and C5:G36 where the former are part of the triple interaction (Figure 2B) within the pseudoknot loop.

Within our crystal structure there is clear electron density corresponding to bound glutamine, and this position lies in the center of the conserved nucleotides (Figure 3B and C). Binding occurs in the major groove of PK, with the amino acid and side chain lying in the plane of the long-range C1:G40 base pair, with the carboxylate group projecting upwards toward the G18 > G2:C39 base triple. The amide side chain is stacked in between C19 and G18 from the ls strand, and the amide itself makes two hydrogen bonds, accepted by the O from C1 N4 and donated by the N to G18 O2′. In the same plane, the amino acid N is hydrogen bonded to G40 O6. One of the carboxylate O atoms of the amino acid is hydrogen bonded to C39 N4 in the G18 > G2:C39 triple within the plane above the C1:G40 base pair.

Adjacent to the bound glutamine, we find additional electron density adjacent to the G18 > G2:C39 triple that corresponds to a bound metal ion (Figure 3C and Supplementary Figure S7). The center lies 2.4 Å from the other carboxylate O atom and is therefore directly bound. Five remaining water molecules forming the inner sphere of hydration can be positioned to make interactions with G18 O6 and N7, and the *pro*R O atoms of A37 and A38 by rotation around the metal carboxylate bond. Four inner-sphere water molecules are involved in interactions with RNA ligands.

### The specificity of ligand binding in the glutamine-II riboswitch

Four of the five heteroatoms of the glutamine ligand are involved in direct bonding interactions with the RNA, and the remaining carboxylate O is directly bonded to the metal ion that is itself bonded to the RNA through its inner-sphere water molecules.

**Figure 4.**
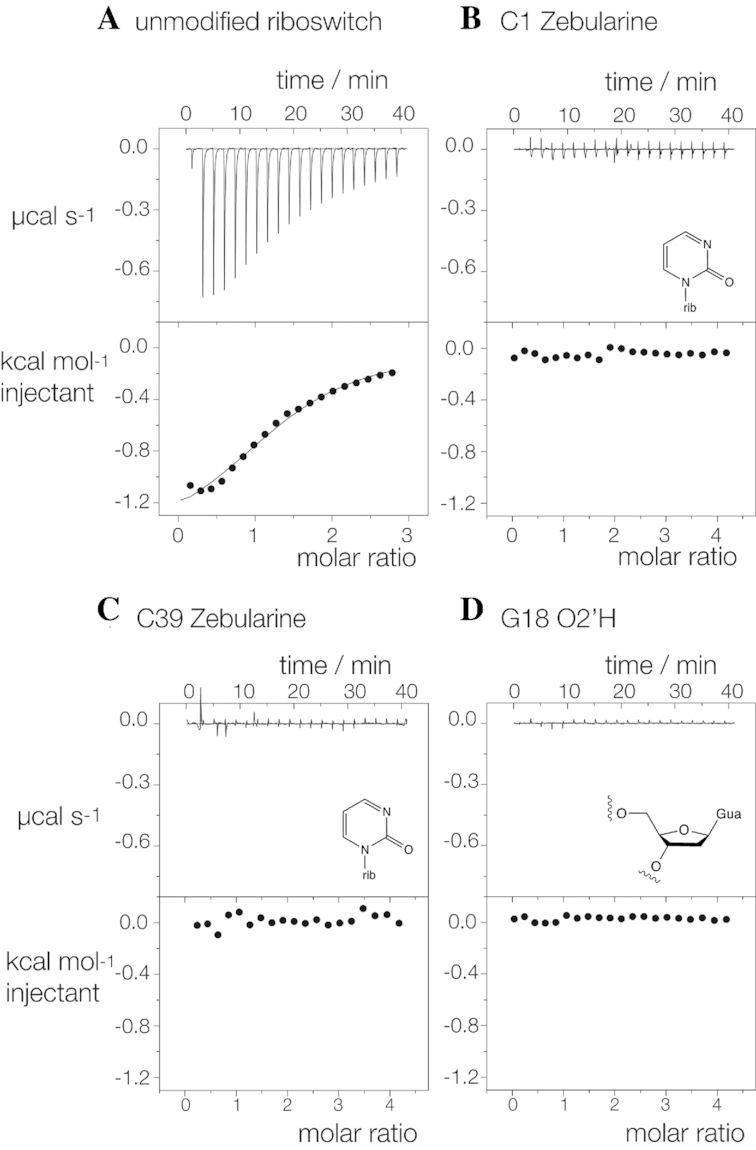
Analysis of the importance of RNA ligands for glutamine binding by atomic mutagenesis and isothermal titration calorimetry. A solution of L-glutamine was titrated into a glutamine-II riboswitch solution, and the heat evolved was measured as the power required to maintain zero temperature difference with a reference cell. Integration over time gives the heat required to maintain thermal equilibrium between cells. In each case, the upper panel shows the raw data for sequential injections of 2 μl volumes (following an initial injection of 0.4 μl) of a 2–4 mM solution of glutamine into 200 μl of a 100–300 μM RNA solution in 40 mM HEPES (pH 7.2), 100 mM KCl, 10 mM MgCl_2_. This represents the differential of the total heat (i.e. enthalpy Δ*H*° under conditions of constant pressure) for each glutamine concentration. Integrated heat data were analyzed using a one-set-of-sites model in MicroCal Origin following the manufacturer’s instructions. The first data point was excluded in the analysis. All ITC experiments were repeated a total of three times. (**A**) Titration of unmodified glutamine-II riboswitch (see Supplementary Table S2). (**B–D**) Titration of glutamine into C1Z (Z = zebularine), C39Z and G18 O2’H modified glutamine-II riboswitch. The relevant chemical structures of the modified nucleotides are shown as inserts. Each substitution will disrupt a contact with glutamine identified in the crystal structure.

We have sought to test the importance of the direct RNA interactions by atomic mutagenesis coupled with calorimetry to measure glutamine affinity (Figure 4 and Supplementary Table S2). The unmodified riboswitch RNA binds L-glutamine in an exothermic reaction, with an affinity of *K*_d_ = 190 μM and a stoichiometry of 1.3. C1 was replaced with zebularine (Z), which lacks the N4 atom (C1Z). No measurable heat was evolved indicative of a complete failure to bind the ligand. This is consistent with disruption of the hydrogen bond to the amide O atom by removal of the donor (Figure 3C). A C39Z atomic mutant was similarly unable to bind glutamine. Removal of C39 N4 will remove both amine protons that are donated to the ligand carboxylate (Figure 3C) and to G2 O6 in the G18 > G2:C39 triple interaction (Figure 2C). Finally, we removed the 2’-hydroxyl group from G18 (G18 O2’H), with the result that this atomic mutant too failed to exhibit measurable binding of glutamine. This is consistent with a requirement for the hydrogen bond donated by the amide N to G18 O2′ (Figure 3C). Thus, all the atomic mutants give results that are fully in accord with the manner of glutamine binding observed in the crystal. When we repeated the titration of the unmodified riboswitch in the absence of Mg^2+^ ions no binding was observed (Supplementary Figure S8). This is consistent with a requirement for the interaction of the L-glutamine ligand with an ion as observed in the crystal, although other explanations are possible for the failure to observe heat evolution.

It is vitally important to the physiological role of riboswitches that they bind their ligands with great specificity, which requires excluding molecules of similar but not identical chemical structure. It would clearly be important that a glutamine riboswitch does not bind glutamate, aspartate or asparagine. The glutamine-II riboswitch binds its ligand with multiple interactions at the amino acid end and at the amide end. Changing the amide to a carboxylate (glutamate or aspartate) would remove the hydrogen bond to G18 O2′H, and we have seen above that this prevents measurable binding of ligand. The dimethylene section of the side chain makes no direct interactions beyond stacking. If this was shortened by one CH_2_ link (asparagine or aspartate), this shortens the distance between the two functional ends by 1.3 Å and reorients them to some degree. The shortened ligand would be unable to make optimal contacts at both ends as it can for glutamine.

### A two-piece form of the glutamine-II riboswitch

**Figure 5.**
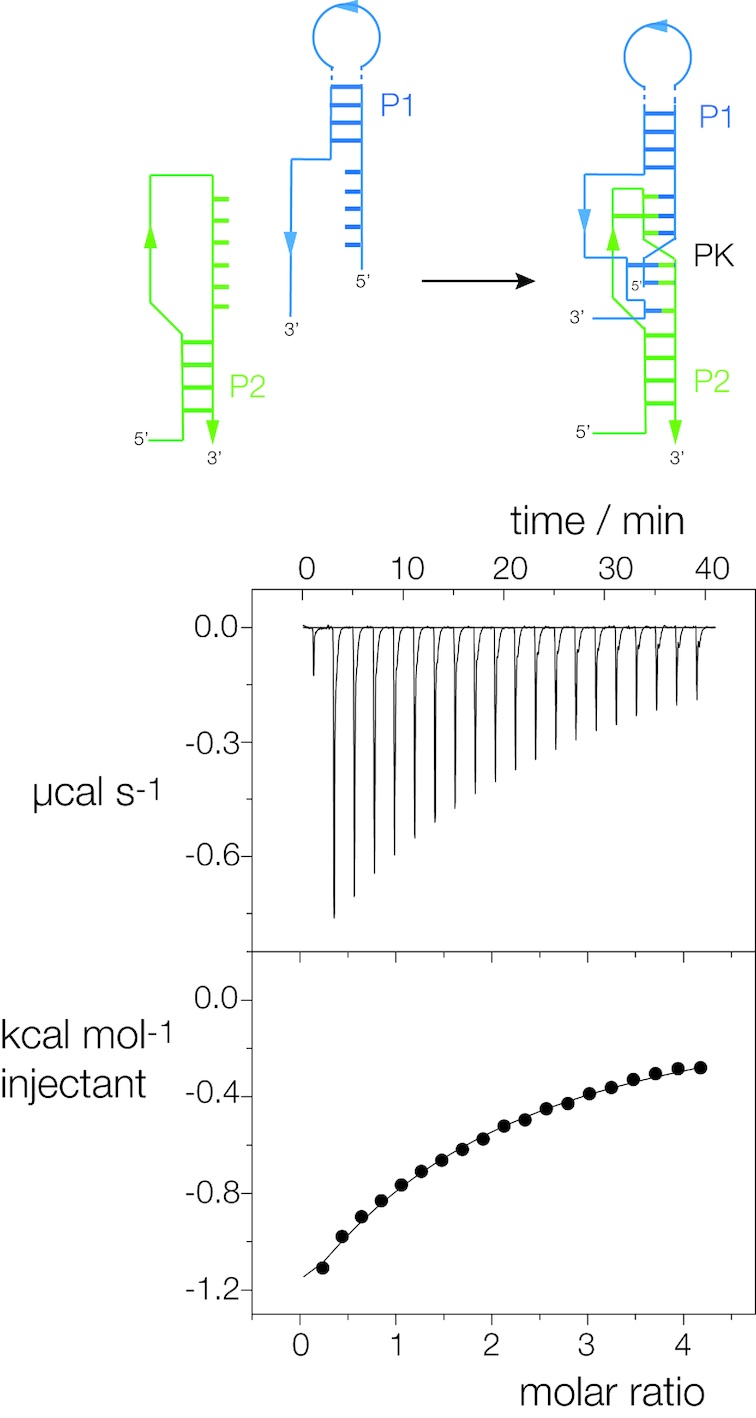
Formation of a functional glutamine-II riboswitch from two covalently unconnected RNA molecules. The schematic shows the association of the two RNA pieces to form a complete riboswitch by formation of the PK helix. This was analyzed by isothermal titration calorimetry. A solution of L-glutamine was titrated into an equimolar mixture of the two RNA species, and the heat evolved was measured as the power required to maintain zero temperature difference with a reference cell as in Figure 4.

The dimeric reassociation of glutamine-II riboswitches in a pairwise manner observed in the crystal suggested that a monomeric riboswitch could be divided into two RNA chains that might be induced to associate through the formation of the PK helix on binding glutamine. We therefore synthesized two RNA oligonucleotides together comprising one complete single riboswitch divided between A20 and U21 in the linker. Glutamine was titrated into an equimolar mixture of the two strands in the microcalorimeter, and exothermic binding was observed (Figure 5). We conclude that glutamine binding stabilizes the interaction of the two RNA species through the formation of the PK region to create a functional ligand binding site.

## DISCUSSION

We have determined the structure of the glutamine-II riboswitch ligand binding domain at 2.3 Å resolution using a novel combination of homology modeling and molecular replacement. The fold of the riboswitch generates three coaxial helical domains, the central one of which is the pseudoknot that has partial triplex character. The L-glutamine ligand binds in the major groove of this helix.

We generally observe that small autonomously folding RNA species are either based around helical junctions or pseudoknot structures. The same is true for creating binding sites for small-molecule ligands. H-type pseudoknot structures tend to form major groove triplexes, and these have been found to create ligand binding sites, for example in the guanidine III (30), preQ1 (31–33) and SAM-II/V (34,35) riboswitches. The glutamine-II riboswitch is clearly an another example of the use of such regions to create binding sites of considerable specificity.

In the SAM-V riboswitch, we have proposed that the key event on binding S-adenosyl methionine ligand is the stabilization of the major groove triplex structure around the ligand binding site (34,35), consistent with patterns of protection in in-line probing experiments (36). It might be anticipated that a similar mechanism could operate in the glutamine-II riboswitch, given that the ligand binding site has significant similarities. Some support is given by the in-line probing data on that riboswitch by Ames and Breaker (9). Those data suggest that even in the complete absence of glutamine, the sections of RNA forming the stems of P1 and P2 are fully formed and so unreactive. However, the regions of RNA corresponding to the pseudoknot PK helix are weakly reactive in the absence of glutamine, becoming completely protected on addition of the ligand. That is consistent with the key event in control being the ligand-induced stabilization of the PK helix, and thus association with the third strand. It is likely that this competes with an alternative structure that otherwise occludes the ribosome binding site, so freeing it up to permit initiation of translation when a sufficient concentration of glutamine is present. The affinity of the riboswitch for glutamine measured in our ITC experiments is not high, in the micromolar region. However to act in metabolic control it is imperative that the RNA switches at the required threshold of glutamine concentration that is determined by the requirements of cellular metabolism. Thus, evolution will have tuned the affinity to match its characteristics to the cellular requirements. Moreover, since our constructs only contain the aptamer domain, the affinity in the cellular context will result from the algebraic sum free energies of ligand binding plus structural rearrangements in all the sections in the complete unit, and this could be different from that of the aptamer domain alone.

It is interesting to compare our structure and manner of ligand binding with that of the glutamine-I riboswitch determined by Ren *et al.* (11). The structures have been superimposed, shown in Supplementary Figure S9A. The glutamine-I riboswitch is based on a three-way helical junction, with no pseudoknot interaction. The third helix (P3 in that structure) projects out to one side and has no equivalent in our structure. Similarly, there is no equivalent of the glutamine-II P2 helix in the glutamine-I structure. However, if the structures are aligned by the P1 stem–loop (P2 in glutamine-I), then it is apparent that the P1 helix of the glutamine-I riboswitch is the equivalent of the PK pseudoknot helix in the glutamine-II riboswitch. It is also terminated by an equivalent long range base pair. Furthermore, the E-loop motif in the glutamine-I riboswitch that forms a turn at the junction superimposes well with the terminal loop that forms the PK pseudoknot helix in the glutamine-II riboswitch. The similarity between the two riboswitches becomes even more striking when we examine the glutamine binding environments, which are virtually identical (Supplementary Figure S9B). The glutamine is hydrogen bonded to O6 and N4 of the G59:C1 base pair (equivalent to G40:C1 in the glutamine-II riboswitch), and to G22 O2′ and C58 N4 in the base triple lying above, which is the equivalent of G18 > G2:C39 in the glutamine-II riboswitch. A hydrated metal ion is also directly bound to the carboxylate O atom in the same manner as in the glutamine-II riboswitch. Thus despite the very different overall architecture of the two riboswitches, based on a three-way junctions versus a pseudoknot structure, the local environment of the ligand binding site and the interactions with glutamine and a hydrated metal ion are essentially the same.

One difference between the two glutamine riboswitches lies in their strand connectivity. In order to take the glutamine-I riboswitch apart it would be necessary completely to disrupt the base pairing. By contrast we have shown that we can divide the glutamine-II riboswitch aptamer domain into two RNA species that associate through the PK helix and its associated base pairing and triple interactions. This situation is somewhat similar to that in the guanidine-II riboswitch that functions by the formation of a ligand-dependent loop–loop interaction creating two guanidine binding sites (37,38). As well as generating insight into the functions of these riboswitches, they have potential applications in the assembly of nanoscale constructions, where ligand-dependent association could be desirable. For the guanidine-II stem–loop, we have recently synthesized conjoined ligands that bind with elevated affinity (39).

In summary, we see how the glutamine-II riboswitch folds to bind its ligand with great selectivity. The overall structure is quite different from that of the glutamine-I riboswitch, and yet the ligand binding site is remarkably similar. This demonstrates the remarkable versatility of RNA structure and its ability to create a framework for the selective binding of small molecules.

## DATA AVAILABILITY

Atomic coordinates and structure factors have been deposited with the Protein Data bank with accession code 6QN3.

## Supplementary Material

gkz539_Supplemental_File

## References

[1] Roth A., Breaker R.R. The structural and functional diversity of metabolite-binding riboswitches. Ann. Rev. Biochem. 2009; 78:305–334. 19298181 10.1146/annurev.biochem.78.070507.135656 PMC5325118

[2] Serganov A., Nudler E. A decade of riboswitches. Cell. 2013; 152:17–24. 23332744 10.1016/j.cell.2012.12.024 PMC4215550

[3] Sherwood A.V., Henkin T.M. Riboswitch-mediated gene regulation: Novel RNA architectures dictate gene expression responses. Ann. Rev. Microbiol. 2016; 70:361–374. 27607554 10.1146/annurev-micro-091014-104306

[4] Leigh J.A., Dodsworth J.A. Nitrogen regulation in bacteria and archaea. Ann. Rev. Microbiol. 2007; 61:349–377. 17506680 10.1146/annurev.micro.61.080706.093409

[5] van Heeswijk W.C., Westerhoff H.V., Boogerd F.C. Nitrogen assimilation in *Escherichia coli*: putting molecular data into a systems perspective. Microbiol. Molec. Biol. Rev. 2013; 77:628–695. 24296575 10.1128/MMBR.00025-13 PMC3973380

[6] Stadtman E.R. The story of glutamine synthetase regulation. J. Biol. Chem. 2001; 276:44357–44364. 11585846 10.1074/jbc.R100055200

[7] Garcia-Dominguez M., Reyes J.C., Florencio F.J. Glutamine synthetase inactivation by protein-protein interaction. Proc. Natl. Acad. Sci. U.S.A. 1999; 96:7161–7166. 10377385 10.1073/pnas.96.13.7161 PMC22038

[8] Garcia-Dominguez M., Reyes J.C., Florencio F.J. NtcA represses transcription of gifA and gifB, genes that encode inhibitors of glutamine synthetase type I from *Synechocystis sp*. PCC 6803. Mol. Microbiol. 2000; 35:1192–1201. 10712699 10.1046/j.1365-2958.2000.01789.x

[9] Ames T.D., Breaker R.R. Bacterial aptamers that selectively bind glutamine. RNA Biol. 2011; 8:82–89. 21282981 10.4161/rna.8.1.13864 PMC3127080

[10] Klahn S., Bolay P., Wright P.R., Atilho R.M., Brewer K.I., Hagemann M., Breaker R.R., Hess W.R. A glutamine riboswitch is a key element for the regulation of glutamine synthetase in cyanobacteria. Nucleic Acids Res. 2018; 46:10082–10094. 30085248 10.1093/nar/gky709 PMC6212724

[11] Ren A., Xue Y., Peselis A., Serganov A., Al-Hashimi H.M., Patel D.J. Structural and dynamic basis for low-affinity, high-selectivity binding of l-glutamine by the glutamine riboswitch. Cell Rep. 2015; 13:1800–1813. 26655897 10.1016/j.celrep.2015.10.062 PMC4690532

[12] Beaucage S.L., Caruthers M.H. Deoxynucleoside phosphoramidites - a new class of key intermediates for deoxypolynucleotide synthesis. Tetrahedron Lett. 1981; 22:1859–1862.

[13] Wilson T.J., Zhao Z.-Y., Maxwell K., Kontogiannis L., Lilley D.M.J. Importance of specific nucleotides in the folding of the natural form of the hairpin ribozyme. Biochemistry. 2001; 40:2291–2302. 11329299 10.1021/bi002644p

[14] Hakimelahi G.H., Proba Z.A., Ogilvie K.K. High yield selective 3'-silylation of ribonucleosides. Tetrahedron Lett. 1981; 22:5243–5246.

[15] Perreault J.-P., Wu T., Cousineau B., Ogilvie K.K., Cedergren R. Mixed deoxyribo- and ribooligonucleotides with catalytic activity. Nature. 1990; 344:565–567. 2181322 10.1038/344565a0

[16] Winter G., Waterman D.G., Parkhurst J.M., Brewster A.S., Gildea R.J., Gerstel M., Fuentes-Montero L., Vollmar M., Michels-Clark T., Young I.D. et al. DIALS: implementation and evaluation of a new integration package. Acta Crystallogr. D Struct. Biol. 2018; 74:85–97. 29533234 10.1107/S2059798317017235 PMC5947772

[17] Karplus P.A., Diederichs K. Linking crystallographic model and data quality. Science. 2012; 336:1030–1033. 22628654 10.1126/science.1218231 PMC3457925

[18] McCoy A.J., Grosse-Kunstleve R.W., Adams P.D., Winn M.D., Storoni L.C., Read R.J. Phaser crystallographic software. J. Appl. Crystallogr. 2007; 40:658–674. 19461840 10.1107/S0021889807021206 PMC2483472

[19] Mathews B.W. Solvent content of protein crystals. J. Mol. Biol. 1968; 33:491–497. 5700707 10.1016/0022-2836(68)90205-2

[20] Kantardjieff K.A., Rupp B. Matthews coefficient probabilities: Improved estimates for unit cell contents of proteins, DNA, and protein-nucleic acid complex crystals. Protein Sci. 2003; 12:1865–1871. 12930986 10.1110/ps.0350503 PMC2323984

[21] Emsley P., Lohkamp B., Scott W.G., Cowtan K. Features and development of Coot. Acta Cryst. D. 2010; 66:486–501. 20383002 10.1107/S0907444910007493 PMC2852313

[22] Joosten R.P., Long F., Murshudov G.N., Perrakis A. The PDB_REDO server for macromolecular structure model optimization. IUCrJ. 2014; 1:213–220. 10.1107/S2052252514009324 PMC4107921 25075342

[23] Chen V.B., Arendall W.B. 3rd, Headd J.J., Keedy D.A., Immormino R.M., Kapral G.J., Murray L.W., Richardson J.S., Richardson D.C. MolProbity: all-atom structure validation for macromolecular crystallography. Acta Cryst. D. 2010; 66:12–21. 20057044 10.1107/S0907444909042073 PMC2803126

[24] Watkins A.M., Geniesse C., Kladwang W., Zakrevsky P., Jaeger L., Das R. Blind prediction of noncanonical RNA structure at atomic accuracy. Sci. Adv. 2018; 4:eaar5316. 29806027 10.1126/sciadv.aar5316 PMC5969821

[25] Waterhouse A.M., Procter J.B., Martin D.M.A., Clamp M., Barton G.J. Jalview Version 2 - a multiple sequence alignment editor and analysis workbench. Bioinformatics. 2009; 25:1189–1191. 19151095 10.1093/bioinformatics/btp033 PMC2672624

[26] Molinaro M., Tinoco I. Use of ultra stable UNCG tetraloop hairpins to fold RNA structures: Thermodynamic and spectroscopic applications. Nucleic Acids Res. 1995; 23:3056–3063. 7544890 10.1093/nar/23.15.3056 PMC307149

[27] Trausch J.J., Marcano-Velázquez J.G., Matyjasik M.M., Batey R.T. Metal ion-mediated nucleobase recognition by the ZTP riboswitch. Chem. Biol. 2015; 22:829–837. 26144884 10.1016/j.chembiol.2015.06.007 PMC4557640

[28] Suslov N.B., DasGupta S., Huang H., Fuller J.R., Lilley D.M., Rice P.A., Piccirilli J.A. Crystal structure of the Varkud satellite ribozyme. Nat. Chem. Biol. 2015; 11:840–846. 26414446 10.1038/nchembio.1929 PMC4618023

[29] Ouellet J., Byrne M., Lilley D.M.J. Formation of an active site in trans by interaction of two complete Varkud Satellite ribozymes. RNA. 2009; 15:1822–1826. 19703941 10.1261/rna.1759009 PMC2743049

[30] Huang L., Wang J., Wilson T.J., Lilley D.M.J. Structure of the Guanidine III Riboswitch. Cell Chem. Biol. 2017; 24:1407–1415. 28988949 10.1016/j.chembiol.2017.08.021 PMC5696562

[31] Klein D.J., Edwards T.E., Ferré-D’Amaré A.R. Cocrystal structure of a class I preQ1 riboswitch reveals a pseudoknot recognizing an essential hypermodified nucleobase. Nat. Struct. Mol. Biol. 2009; 16:343–344. 19234468 10.1038/nsmb.1563 PMC2657927

[32] Spitale R.C., Torelli A.T., Krucinska J., Bandarian V., Wedekind J.E. The structural basis for recognition of the PreQ0 metabolite by an unusually small riboswitch aptamer domain. J. Biol. Chem. 2009; 284:11012–11016. 19261617 10.1074/jbc.C900024200 PMC2670106

[33] Zhang Q., Kang M., Peterson R.D., Feigon J. Comparison of solution and crystal structures of preQ1riboswitch reveals calcium-induced changes in conformation and dynamics. J. Am. Chem. Soc. 2011; 133:5190–5193. 21410253 10.1021/ja111769g PMC3085290

[34] Gilbert S.D., Rambo R.P., Van Tyne D., Batey R.T. Structure of the SAM-II riboswitch bound to S-adenosylmethionine. Nat. Struct. Mol. Biol. 2008; 15:177–182. 18204466 10.1038/nsmb.1371

[35] Huang L., Lilley D.M.J. Structure and ligand binding of the SAM-V riboswitch. Nucleic Acids Res. 2018; 46:6869–6879. 29931337 10.1093/nar/gky520 PMC6061858

[36] Poiata E., Meyer M.M., Ames T.D., Breaker R.R. A variant riboswitch aptamer class for S-adenosylmethionine common in marine bacteria. RNA. 2009; 15:2046–2056. 19776155 10.1261/rna.1824209 PMC2764483

[37] Huang L., J. W., Lilley D.M.J. The structure of the guanidine-II riboswitch. Cell Chem. Biol. 2017; 24:695–702. 28529131 10.1016/j.chembiol.2017.05.014 PMC5486947

[38] Reiss C.W., Strobel S.A. Structural basis for ligand binding to the guanidine-II riboswitch. RNA. 2017; 23:1338–1343. 28600356 10.1261/rna.061804.117 PMC5558903

[39] Huang L., Wang J., Wilson T.J., Lilley D.M.J. Structure-guided design of a high affinity ligand for a riboswitch. RNA. 2019; 25:423–430. 30609994 10.1261/rna.069567.118 PMC6426286

