# Supplementary material for: Structure and ligand binding of the glutamine-II riboswitch
Source: Nucleic Acids Res. 2019 Jun 19;47(14):7666–75. doi: 10.1093/nar/gkz539 (PMC6698751; doi:10.1093/nar/gkz539)
Supplement: gkz539_Supplemental_File [file gkz539_supplemental_file.pdf]

Structure and ligand binding of the glutamine II riboswitch

L. Huang, J. Wang, A. M. Watkins, R. Das and D. M. J. Lilley

SUPPLEMENTARY FIGURES

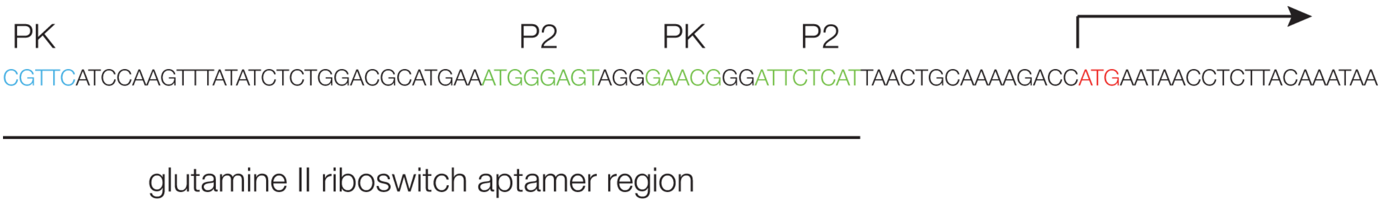

Figure S1. The location of the riboswitch aptamer domain relative to the start codon for translation for Prochlorococcus sp RS01_CP018345.1. The riboswitch begins at PK and runs through to P2. The ATG corresponding to the initiator codon is shown in red.

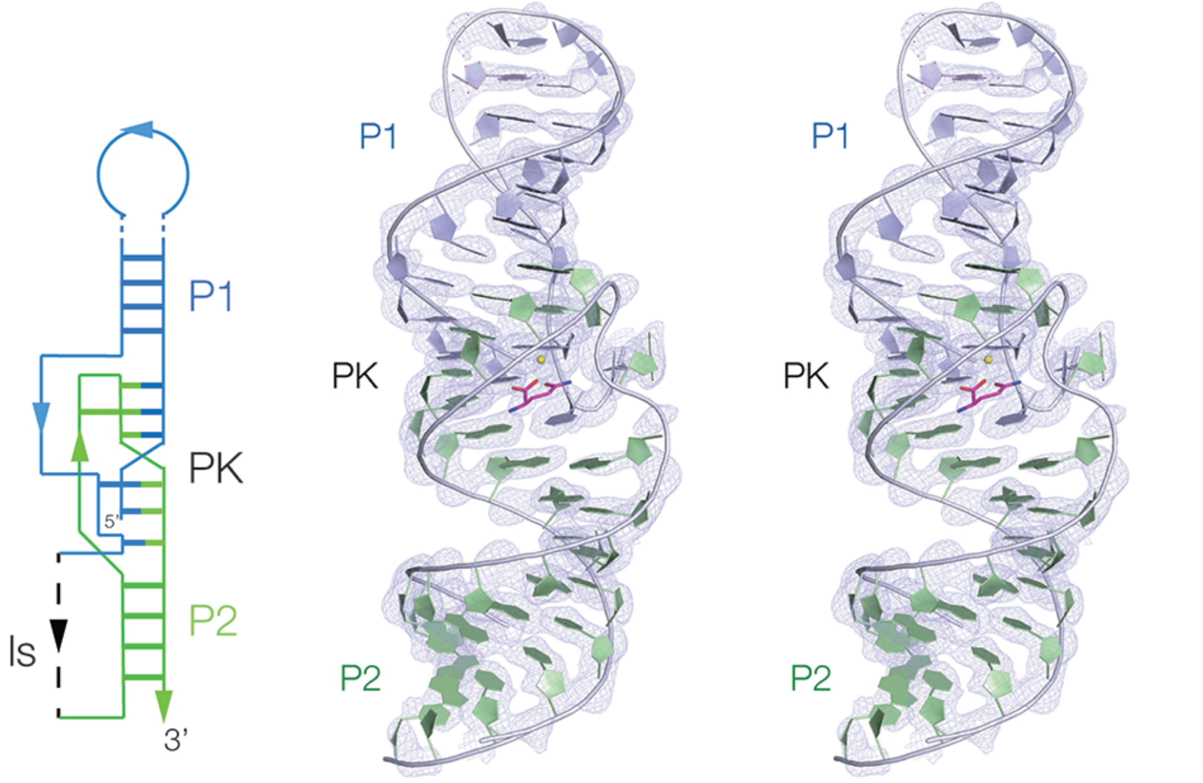

Figure S2. Composite omit map ($2mF_o$-DF_c) of a complete functional unit of the glutamine-II riboswitch, contoured at 1.5σ. The glutamine ligand is shown in stick form, colored magenta. A parallel-eye stereoscopic image is shown.

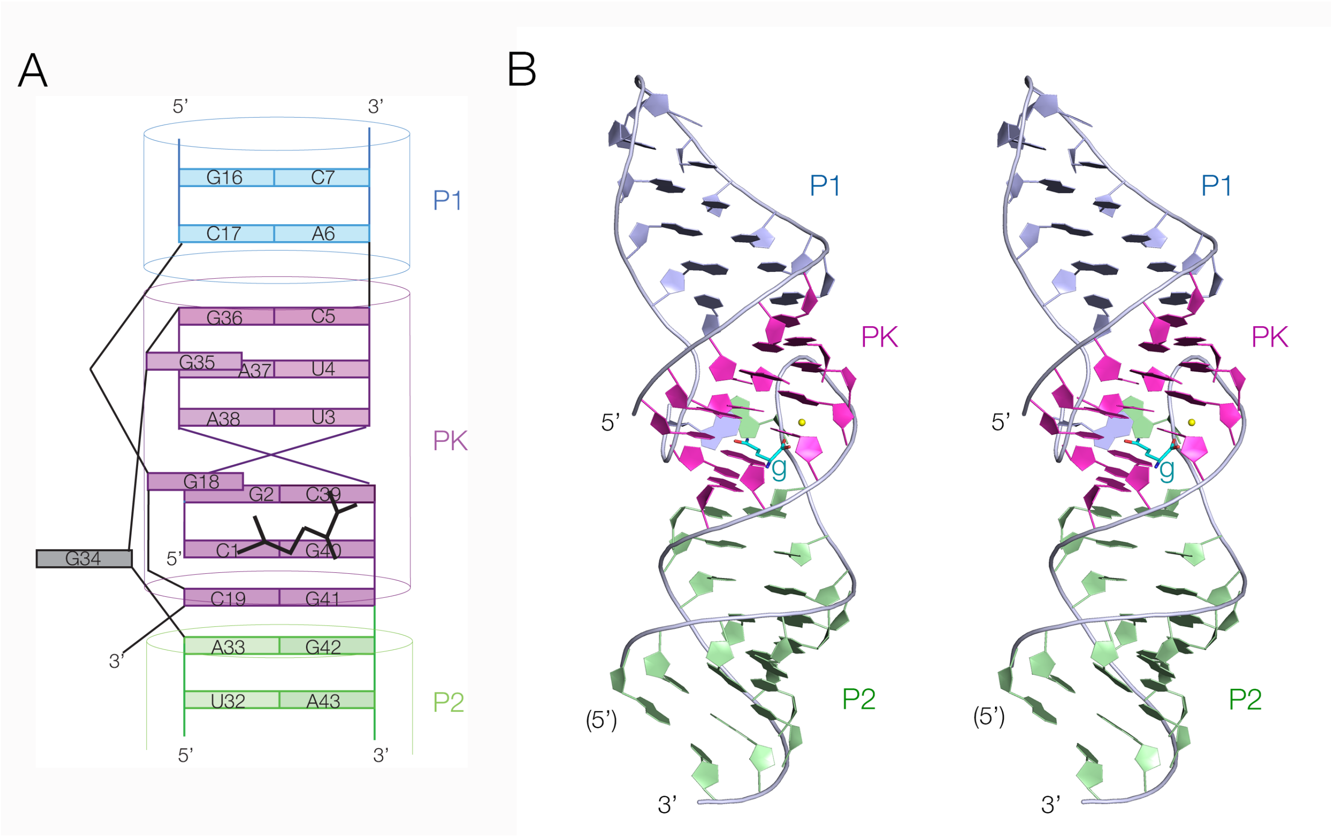

Figure S3. The functional unit of the glutamine-II riboswitch colored to highlight the three coaxially stacked helices. In this figure the hybrid helix PK is colored magenta.

A. Schematic illustration of the structure of the functional core of the riboswitch. The bound glutamine molecule is indicated in black.

B. The structure of the complete riboswitch functional unit, shown in parallel-eye stereoscopic representation. The glutamine (g) is shown in stick form, colored cyan.

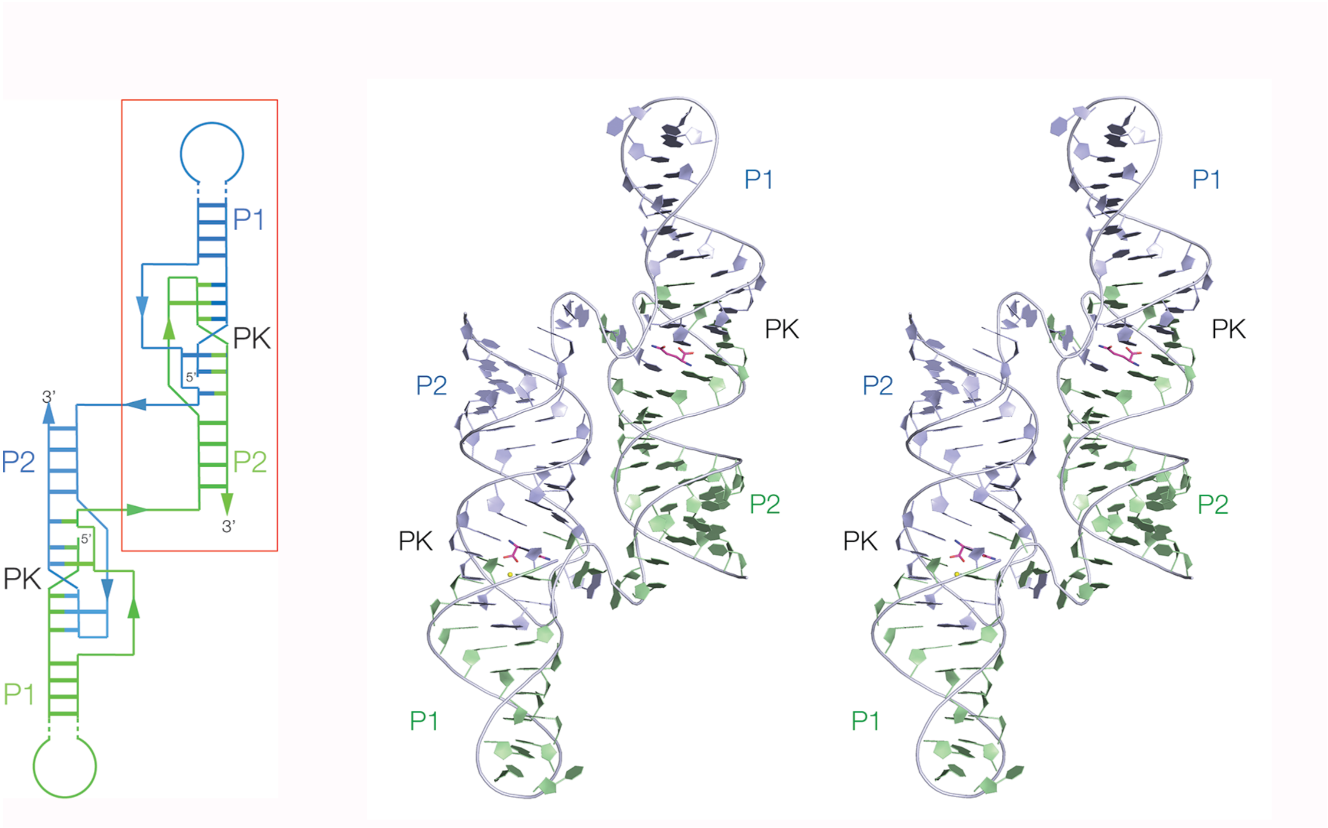

Figure S4. The structure of the asymmetric unit in the crystal of the glutamine-II riboswitch, comprising two composite functional units of the riboswitch. A parallel-eye stereoscopic image is shown.

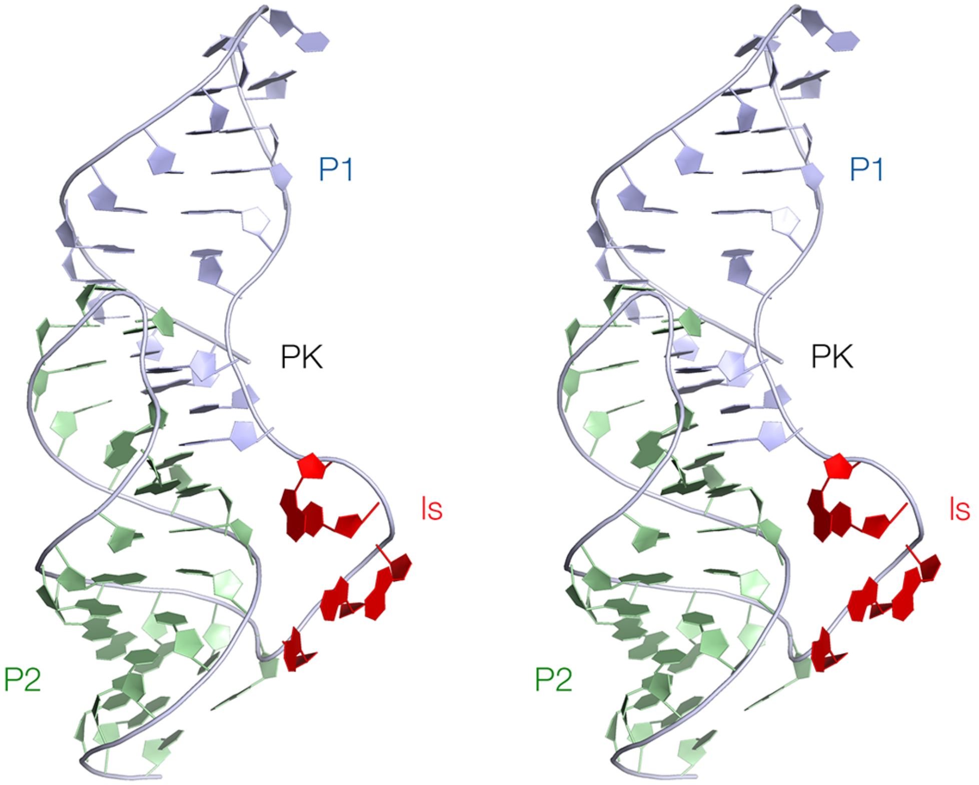

Figure S5. A parallel-eye stereoscopic image of a model of the probable monomeric structure of the glutamine-II riboswitch. The linking strand (ls) is colored red.

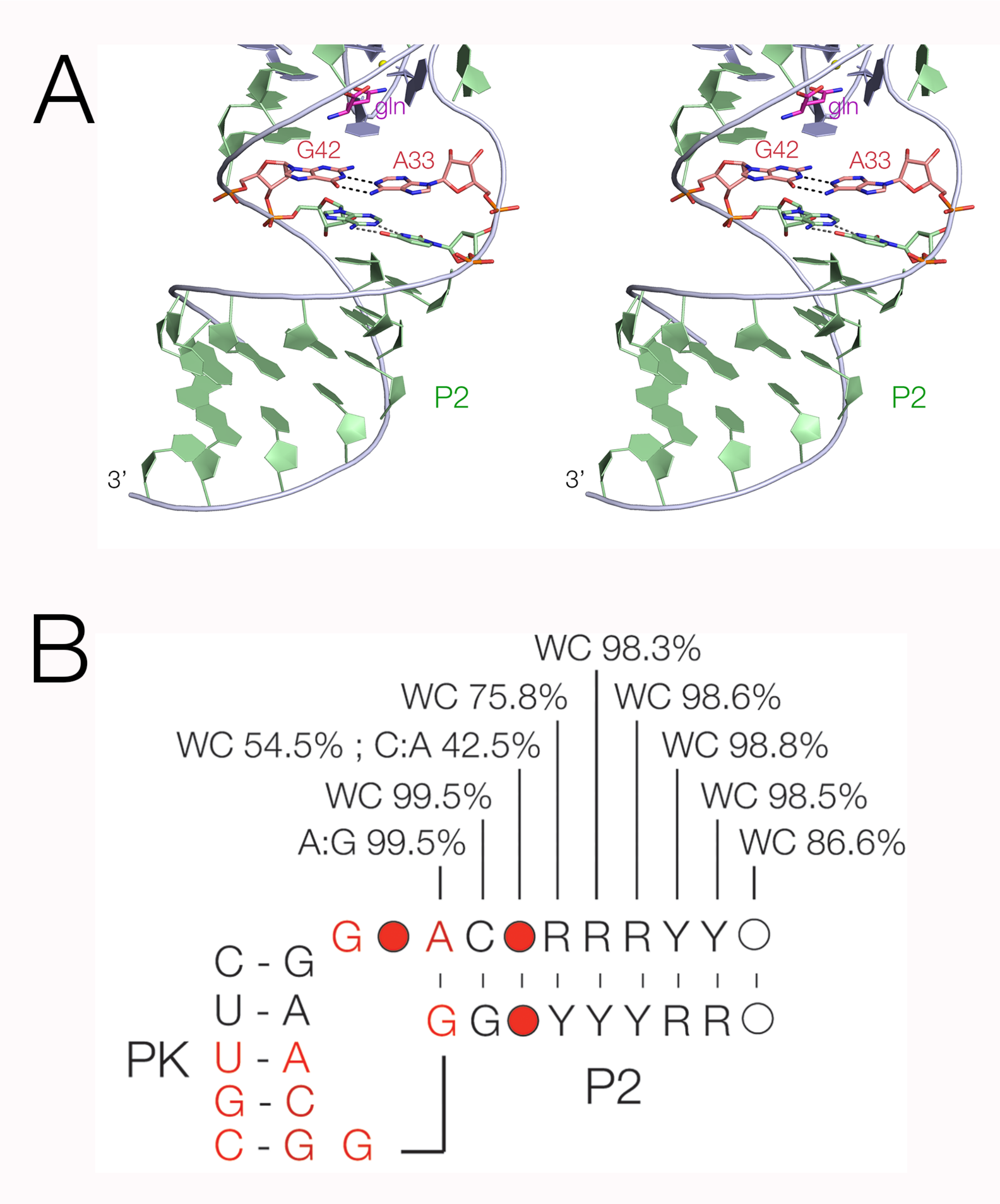

Figure S6. Realignment of the P2 helix in the light of the crystal structure of the glutamine-II riboswitch.
A. The P2 helix, with the A33:G42 *cis*-Watson-Crick base pair at the end of P2 adjoining the PK helix highlighted red. A parallel-eye stereoscopic image is shown.
B. Realignment of the base pairing of P2. The % conservation of each base pair is shown. WC = Watson-Crick base pair.

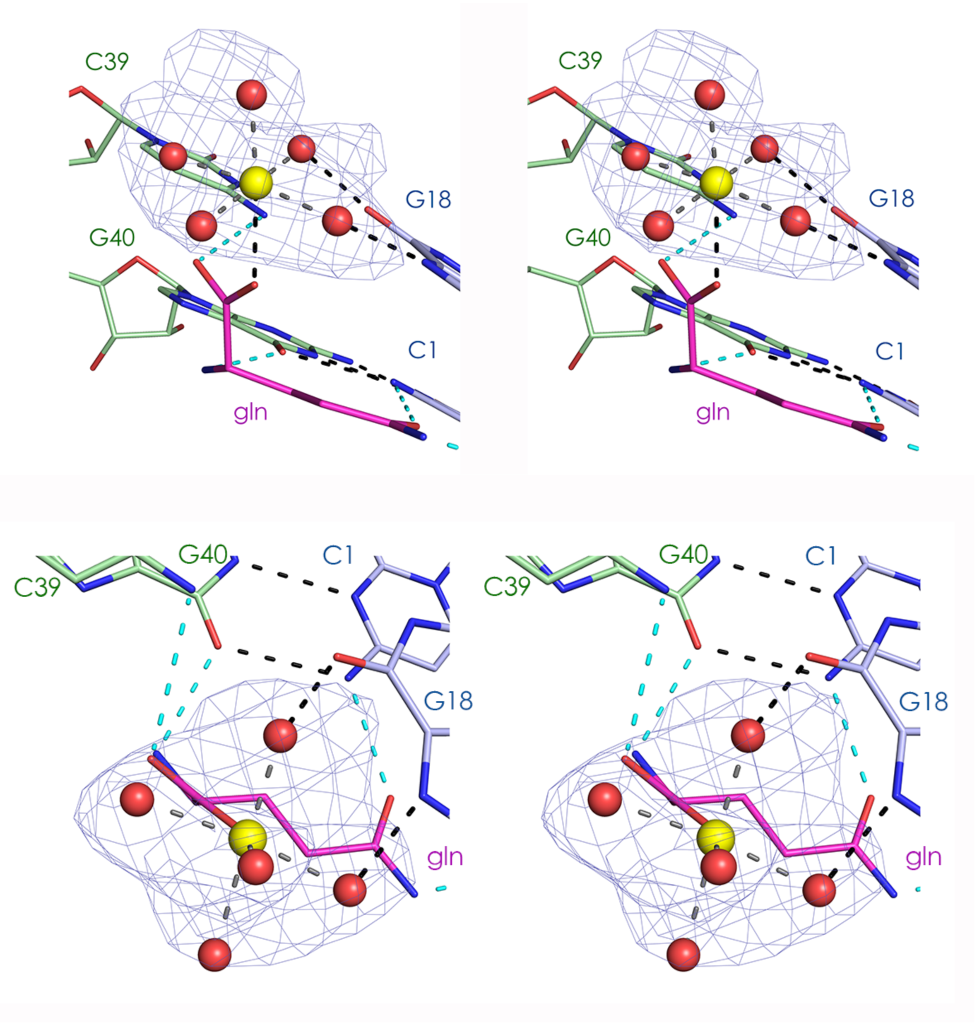

Figure S7. A hydrated metal ion in the glutamine binding site of the glutamine-II riboswitch. The electron density shows an omit map (mF_o-DF_c generated in Phenix) generated by omitting the water molecules hydrating the metal ion contoured at 3 σ. Parallel-eye stereoscopic images are shown from two perspectives; side-on (upper) and along (lower) the metal-carboxylate bond. Hydrogen bonds between the glutamine ligand and the RNA are highlighted cyan. The metal ion is shown as the yellow sphere, and water molecules belonging to the inner sphere of hydration as red spheres.

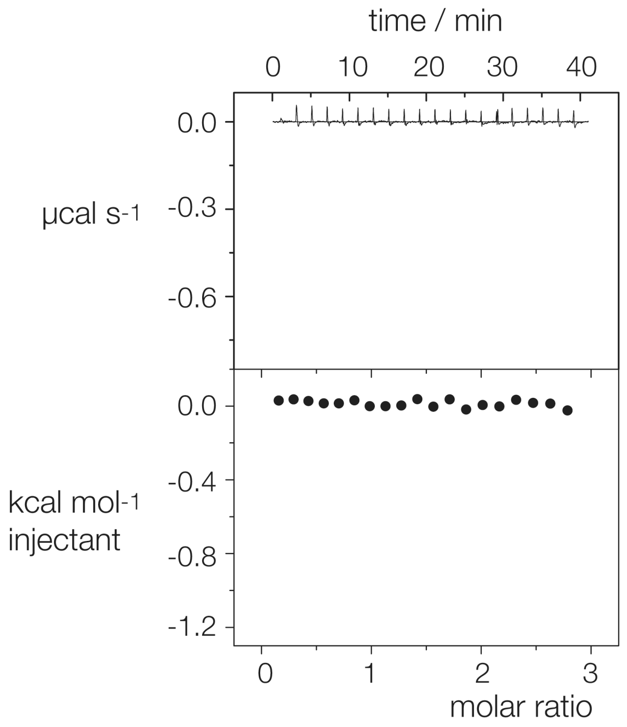

Figure S8. Study of binding of L-glutamine to unmodified glutamine-II riboswitch RNA in the absence of divalent cations analysed by isothermal titration calorimetry. The titration was performed in the equivalent manner to that in Figure 4. The upper panel shows the raw data for sequential injections of 2 µL volumes (following an initial injection of 0.4 µL) of a 4 mM solution of L-glutamine into 200 µL of a 300 µM RNA solution in 40 mM HEPES (pH 7.2), 100 mM KCl.

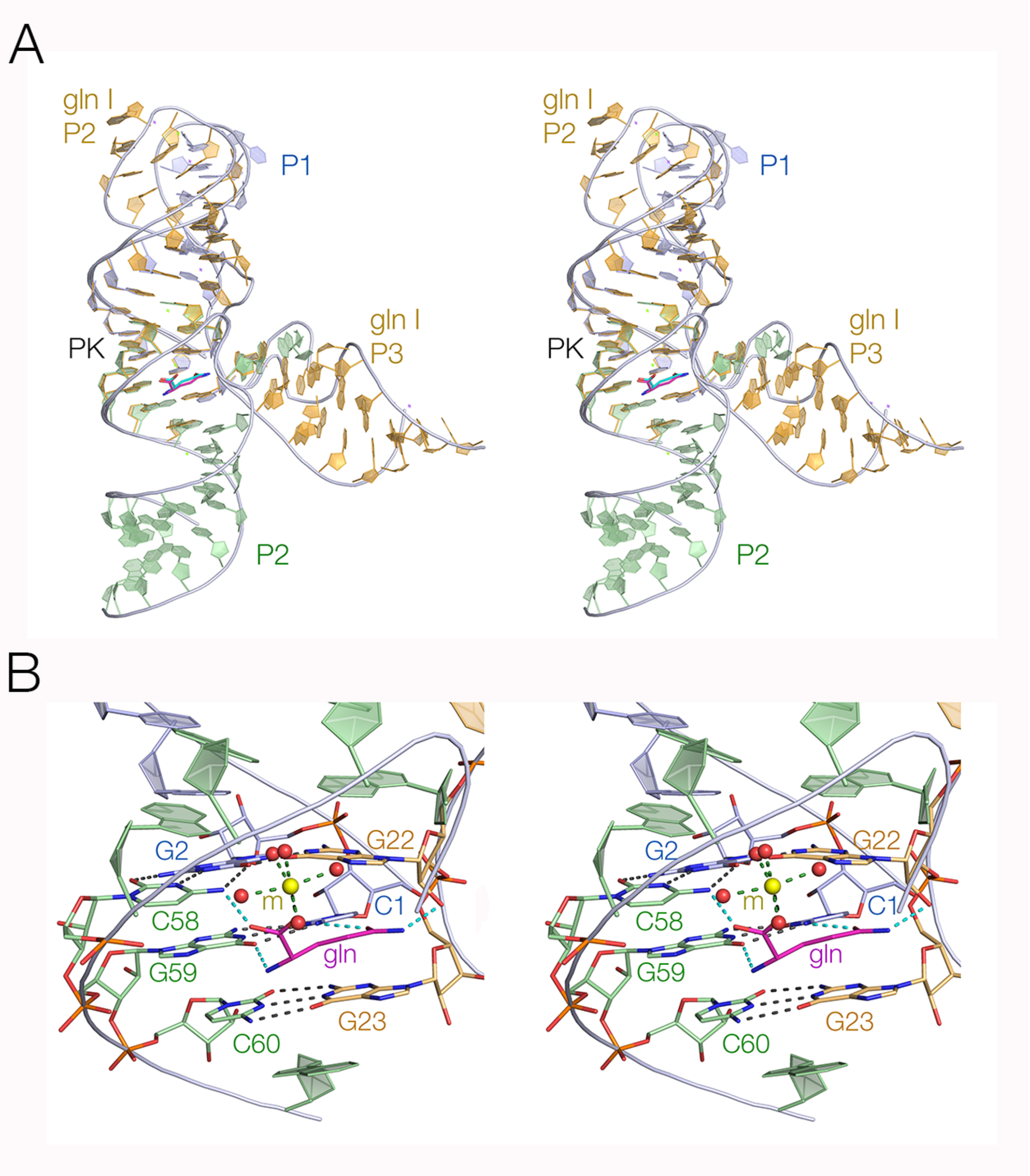

Figure S9. Comparison with the glutamine-I riboswitch structure (Ren et al, *Cell Rep*, **13**, 1800-1813, 2015). Parallel-eye stereoscopic images are shown.

A. Superposition of the glutamine-II and I riboswitches. For both riboswitches the RNA is shown in cartoon form, and the glutamine ligand in stick form. For the glutamine-I riboswitch the ligand is colored magenta, and for the glutamine-II riboswitch the ligand is colored cyan. The glutamine-II riboswitch RNA is colored blue/green as previously, and the glutamine-I riboswitch RNA is colored orange.

B. Glutamine bound to its binding site in the glutamine-I riboswitch, taken from PDB ID 5DDP. Nucleotides are numbered as in the PDB file.

SUPPLEMENTARY TABLES

	DasModel 1	DasModel 2	DasModel 3	DasModel 4	DasModel 5
RMSD (P1, PK and P2)	3.22	3.45	3.59	4.96	4.72
RMSD (PK and P2)	1.78	1.99	1.74	3.09	2.83
LLG (PK and P2)	345	166	291	205	148
TFZ (PK and P2)	14.9	5.9	13.0	6.7	6.1

Table S1. Search models used in molecular replacement and their RMSD values. RMSD (P1, PK and P2) are the RMSD values for the 5 blind models to the structure determined by crystallography. RMSD (PK and P2) are the RMSD values for the 5 blind models to the determined structure using only the PK and P2 parts. LLG (PK and P2) is the overall LLG for two copies of PK and P2 by PHASER. TFZ (PK and P2) is the translation function Z-scores for two copies of PK and P2 by PHASER.

RNA	*n*	ΔH / kcal.mol^{-1}	ΔS / cal.K^{-1}mol^{-1}	ΔG / kcal.mol^{-1}	K_d / μM	c*
wild type 3 replicates	1.3 ± 0.2	-2.1 ± 0.5	9.8 ± 2.6	-6.3 ± 2.4	190 ± 90	1.6

Table S2. Thermodynamic parameters for L-glutamine binding to the unmodified glutamine-II riboswitch. Several of the atomic mutants lead to no measurable evolution of heat on titration of L-glutamine, so that no parameters can be obtained. These are not therefore included in this table. The values are the average of three independent ITC titrations.

* $c = [M]_t \cdot Ka$ An acceptable range is given by $1 < c < 1000$. (Turnbull JACS 2003).
